# Temperament and character traits and profiles: impact on bipolar disorder risk and onset age

**DOI:** 10.3389/fpsyt.2025.1721514

**Published:** 2026-01-06

**Authors:** Ioannis Filis, Androniki Naska, Anastasia Antoniou, Ioanna Constantinidi, Vassiliki Benetou, Panagiotis Ferentinos

**Affiliations:** 1 2^nd^ Department of Psychiatry, School of Medicine, National and Kapodistrian University of Athens, Attikon University Hospital, Athens, Greece; 2 Department of Hygiene, Epidemiology and Medical Statistics, School of Medicine, National and Kapodistrian University of Athens, Athens, Greece

**Keywords:** age of onset, bipolar disorder, character, personality, profiles, temperament

## Abstract

**Objective:**

This study investigated how personality (Temperament, Character) influences the occurrence and onset age of Bipolar Disorder (BD). Unlike previous studies, Temperament and Character Inventory (TCI) dimensions as well as profiles were examined regarding BD onset age.

**Methods:**

We recruited within 5 years 179 adults with prevalent BD (37.4% males, mean age 48.0 ± 12.0 years) attending a general hospital outpatient clinic, in euthymia during the previous 3 months, and 96 controls (36.5% males, mean age 40.4 ± 12.6 years), a convenience sample of community adults recruited via snowball sampling. All participants completed TCI-140. Associations of personality with diagnosis were investigated using logistic regressions while case-only linear and Cox regressions examined associations of personality with onset age. Analyses included TCI dimensions, continuous and binary (dichotomized utilizing Greek general population medians), and profiles (all possible combinations of Temperament or Character high/low binary dimensions), adjusting for potential confounders.

**Results:**

In logistic regressions, BD risk was associated with higher scores in Novelty Seeking (NS), Harm Avoidance (HA) and Self-Transcendence (ST) and lower in Self-Directedness (SD) and Cooperativeness (CO). In linear and Cox regressions, high NS indicated earlier onset compared to low (B=-4.70 [-8.10, -1.30]; HR = 1.60 [1.15, 2.22]), while high SD indicated delayed onset (B = 5.24 [1.87, 8.62]; HR = 0.57 [0.40, 0.79]). In exploratory profile analyses, Narcissistic (high NS, HA and Reward Dependence [RD]) and Histrionic (high NS, low HA, high RD) Temperament profiles and Cyclothymic (low SD, high CO, high ST) and Melancholic (low SD, CO and ST) Character profiles were associated with earlier BD onset, while the Reliable (low NS, low HA, high RD) Temperament profile and Bossy (high SD, low CO, low ST), Creative (high SD, CO and ST) and Organized (high SD, high CO, low ST) Character profiles were associated with delayed onset. These associations, however, lost statistical significance after correcting for multiple comparisons and should be interpreted cautiously.

**Conclusion:**

This study reaffirmed previous associations of TCI dimensions with BD risk, with two (NS, SD) also significantly relating for the first time to onset age. TCI profiles tentatively provided a more nuanced understanding of BD’s onset than dimensions but warrant further investigation in larger samples.

## 1 Introduction

Bipolar Disorder (BD) is one of the leading causes of disability worldwide and poses a significant health burden, with the BD spectrum (the two BD subtypes, BDI and BDII, as well as Cyclothymia) having a lifetime prevalence of more than 6% (1, 2). Though symptoms typically occur at around 20 years of age, they may first appear in adolescence or late childhood (3). Earlier onset is generally associated with worse prognosis, more severe episodes, and higher psychiatric comorbidity (4). Some risk factors for early onset BD include family history of BD, childhood psychiatric comorbidity, prenatal and perinatal events, trauma and other environmental stressors (3, 5–7). Male sex and BDI also appear to be associated with earlier onset as well as worse prognosis (4, 8, 9). While the exact pathophysiologic mechanisms remain unclear, BD results from both genetic influences with a heritability around 60-85% and environmental factors that may play a predisposing or triggering role for the disease (10–15).

Personality and emotional disposition may also predispose to major psychiatric disorders and affect their natural history and prognosis. Kraepelin was the first to link mood disorders to distinct personality types (16–18), revisited in modern times by Akiskal, who attributed to his five temperaments a pathoplastic role for his soft bipolar spectrum (19). More recent theories place dimensional personality pathology at the extreme end of five continua (20) adopted from the Five-factor Model (FFM) of personality (21) but this framework was recently broadened to include all psychopathology along the same basic five continua in a new classification system called the Hierarchical Taxonomy of Psychopathology (HiTOP) (22). Interestingly, a recent meta-analysis sought to expand the temperamental theoretical basis of the HiTOP model by investigating associations of its five spectra and related FFM dimensions with temperament traits of two most influential models, namely Akiskal’s temperaments and Cloninger’s psychobiological model (23).

Claude Robert Cloninger developed his psychobiological personality model to help explain vulnerability to psychiatric disorders, applying biology along with his dimensional approach to personality theory (24, 25). Cloninger linked personality dimensions to specific neurobiological functions, for example low dopaminergic, high serotonergic and low noradrenergic basal activity linked to high Novelty Seeking (NS), Harm Avoidance (HA) and Reward Dependence (RD), respectively (24, 26). Cloninger’s theory distinguished between the Temperament (unconscious, heritable, less influenced by environmental factors) and the Character (conscious and subject to socio-environmental and cultural influences). Temperament was considered the emotional core of self, and relatively stable after adolescence, while Character was theorized to develop well into adulthood, although alterations after the early adult years were considered negligible. NS, HA and RD evolved to comprise the 3 independent Temperament dimensions, along with a fourth one, Persistence (P) (25). NS measures thrill seeking, extraversion, impulsivity, and aversion to routine. HA measures sensitivity to painful or unpleasant stimuli with higher scores linked to introversion, pessimism, passivity, and neuroticism. RD measures response to rewarding stimuli, especially social, with higher scores translating to approval seeking behavior and sentimentality, as well as trust in others. Finally, P represents perseverance despite obstacles, and a high score is associated with determination, perfectionism, and diligence. A recent meta-analysis showed that each TCI temperamental dimension correlates to specific FFM traits: high HA is mainly linked to high neuroticism and low extraversion, high NS to high extraversion and low conscientiousness, high RD to high extraversion and agreeableness, while high P is mainly linked to high conscientiousness (23). Furthermore, Character included 3 separate independent dimensions, Self-Directedness (SD), Cooperativeness (CO) and Self- Transcendence (ST) (25). SD reflects one’s adaptability in pursuit of a goal and ability to delay gratification, and Cloninger described it as pivotal to the presence or absence of personality disorder (PD). High SD is linked to high self-esteem, responsibility, and resourcefulness. Meanwhile, CO evaluates social tolerance, agreeability, and empathy, with higher scores indicating kindness, empathy, and strong communal bonds, while low scores suggest distrust and hostility. ST measures spirituality, faith, altruism, and sense of unity, with low scores indicating materialism and cynicism and high scores suggesting a sense of meaning and idealism. These 3 dimensions reflect an individual’s sense of self, others, and the universe as a whole, respectively (27).

The Temperament and Character Inventory (TCI), coming in three versions with different numbers of items or scoring procedures, is the tool devised by Cloninger to measure the 7 dimensions proposed in his psychobiological model (25, 28). Apart from numeral dimensions (total continuous scores on each TCI dimension), TCI also offers binary (dichotomized) dimensions, i.e., “high” and “low” scores based on median values from a representative general population sample. Furthermore, Cloninger encouraged the use of specific TCI profiles, i.e., complex interactions between triads of specific dimensions. TCI profiles are schematically hosted in the Temperament, Character, and Resilience cubes. Each vertex of a cube reflects one of 8 different Temperament, Character, and Resilience profiles. The 8 profiles of each cube result from all possible combinations of 3 high/low dichotomized dimensions: high or low NS, HA and RD for the Temperament cube; high or low SD, CO and ST for the Character cube; and finally high or low HA, P and SD for the Resilience Cube (27).

Since its introduction, TCI has been widely investigated in both clinical and non-clinical contexts. In a recent comprehensive meta-analysis exploring associations between TCI dimensions and various psychopathological conditions, high HA and low SD were identified as the core traits associated with psychopathology, including BD (29). High NS, low CO, and high ST also had weaker associations with BD. Another earlier meta-analysis focusing specifically on mood disorders recorded similar associations between TCI dimensions and BD and additionally compared BDI and BDII subgroups, without detecting any significant differences among them (30). Both meta-analyses reported effects with considerable heterogeneity, which might be explained by different study or sample characteristics, such as mood state at assessment, psychiatric family history, disease stage, treatment, and comorbid personality or psychiatric disorders (29, 30). Another source of heterogeneity arises from the fact that, as suggested by Cloninger (27), the association of TCI dimensions with risk is only conditional on other dimensions, i.e., TCI profiles rather than dimensions themselves are directly associated with the disease. Yet, studies investigating specific TCI profiles in relation to BD and its subtypes are lacking.

To prioritize TCI dimensions in terms of their clinical impact in BD, we previously explored their associations with patients’ self-reported resilience (31). A more objective prognostic index of the disease is age at onset (AAO), with earlier onset predicting a less benign course. Yet, to the author’s knowledge, only one study has investigated TCI dimensions and BD onset, among various other clinical features, yielding no significant findings (32). That multi-site cross-sectional study assessed a heterogeneous multinational sample of patients with mood disorders and healthy blood donors using two TCI versions; almost 50% of patients were inpatients and almost 80% were depressed at intake. The authors noted that sample heterogeneity, which they tried to control by adjusting for inpatient setting, might limit the study’s validity.

In summary, although TCI dimensions have been previously associated with BD, their relationship with BD AAO is scarcely explored (investigated in only one study with potential limitations). Lastly, TCI profiles remain unexplored in relation to both BD risk and its AAO. Given these gaps in current literature, the aims of this study were to assess how TCI traits and profiles may relate with BD’s occurrence or AAO. The first objective was to evaluate whether specific TCI traits and profiles are associated with BD risk. To that end, comparisons between patients with BD and healthy controls (i.e., not suffering from BD and other major psychiatric disorders) were implemented. The second objective was to assess whether AAO of patients with BD relates to specific TCI traits or profiles.

## 2 Materials and methods

### 2.1 Study sample

For the purposes of this study, data were collected from consenting outpatients followed up at the Affective Disorders and Suicidality Outpatient Clinic of the 2^nd^ Department of Psychiatry, National and Kapodistrian University of Athens at Attikon University Hospital, between November 2018 and October 2023. All patients had received a diagnosis of BD prior to follow-up, and most (90.5%) had been previously hospitalized for a mood episode. Therefore, the patient sample consisted exclusively of prevalent cases.

Experienced psychiatrists and psychologists in the field of mood disorders confirmed diagnosis according to the Diagnostic and Statistical Manual of Mental Disorders – 5^th^ edition (DSM-5) criteria at the initiation of follow-up and reaffirmed it repeatedly thereafter. When there was diagnostic disagreement, cases were discussed until team consensus was reached.

The control sample was collected via snowball sampling; clinic staff reached out to acquaintances from the community who contacted their own acquaintances in turn.

Inclusion criteria required all participants to be adult (≥18 years). Patients were required a confirmed BD diagnosis, and documented euthymic status for at least 3 consecutive months prior to inclusion, as recorded with a Young Mania Rating Scale (YMRS) score of <10 and a Hamilton Depression Rating Scale (HDRS) score of <8 (33, 34). This ascertained that patients’ TCI responses were not influenced by mood symptoms that could potentially alter perception of self and others.

Exclusion criteria for all participants included neurocognitive disorders (delirium, dementia, traumatic brain injury etc.), intellectual disability (IQ<70), lack of fluency in Greek, and less than 6 years of educational attainment (incomplete primary school education). Additional exclusion criteria for patients included severe comorbid psychiatric disorders (alcohol and substance use disorder during the last 6 months, severe PD that would interfere with participation in the study), and for controls a personal or family history (in first-degree relatives) of major psychiatric disorder. No matching criteria were applied between patients and controls.

Data collected from participants included information on age, sex, educational attainment (in years), employment status, profession, and family status. For patients, clinical characteristics (including AAO) and family history of major psychiatric disorder in first-degree relatives were also collected through interviews with patients/informants or extracted from medical files.

All participants provided written informed consent after receiving information about the nature of the study and issues of confidentiality. The study was approved by Attikon hospital research ethics committee under protocol number ΨΥΧ, ΕΒΔ654/01-10-2018.

### 2.2 Personality assessment

TCI originally had 240 items in the form of true-or-false sentences, and a subsequent revised version, TCI-R, also with 240 items, employed a 5-point Likert scale instead. A subsequent shorter version, TCI-140, including 140 items also rated on a 5-point scale, from 1 (“strongly disagree”) to 5 (“strongly agree”), was more appealing for use in clinical environments (35) and was the one selected for this study. Each TCI-140 statement corresponds to one of 7 personality dimensions, 4 for Temperament (NS, HA, RD, and P) and 3 for Character (SD, CO, and ST). All dimensions are evaluated via 20 statements (score 20-100), except ST which is graded via 16 statements (score 16-80). Additionally, 65 items are scored inversely; a “strongly agree” response relates to a score of 1 and vice versa. There are also 4 validity items to ensure reliable responses.

**Figure 1:**
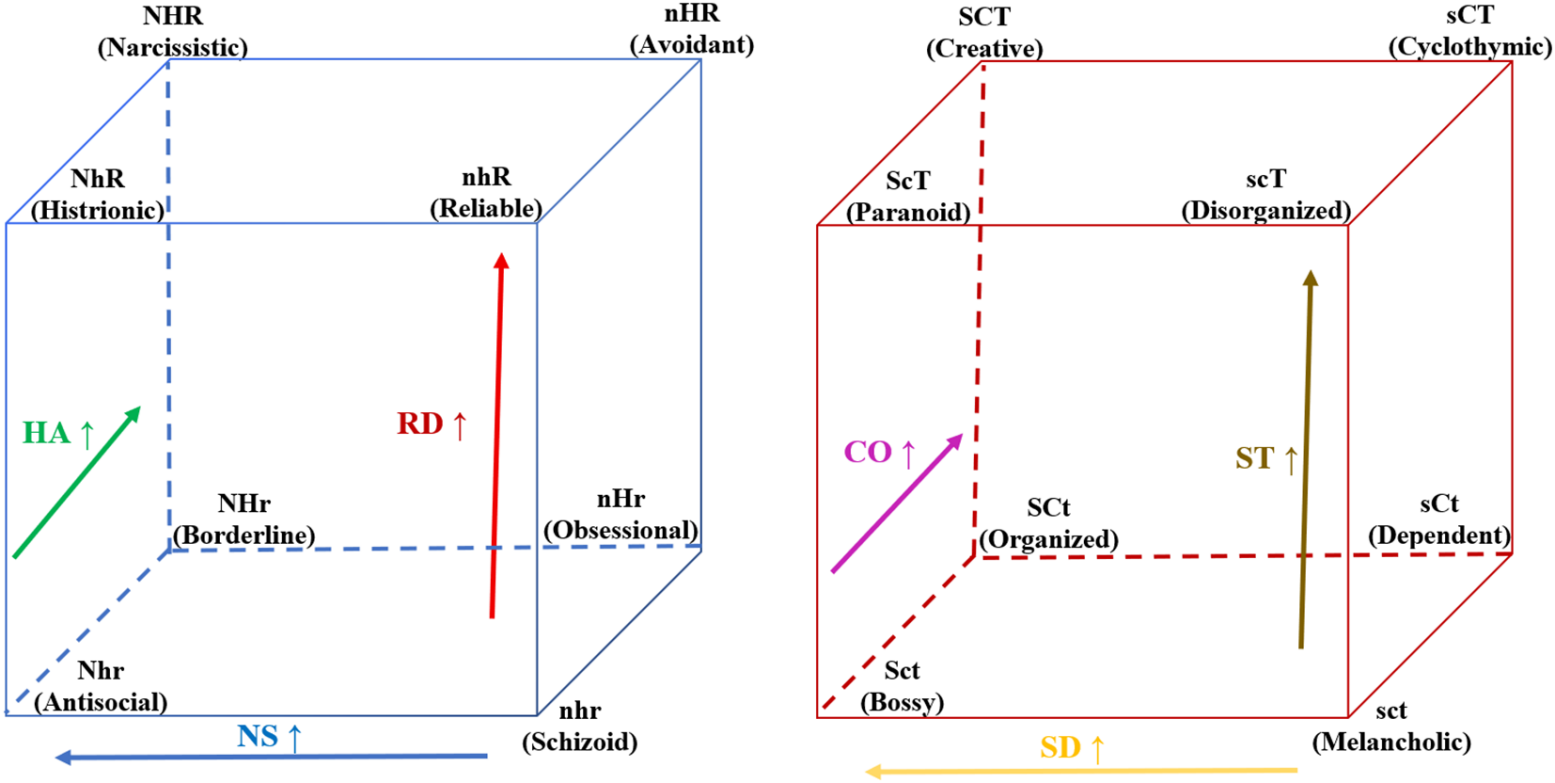
Cloninger’s Temperament (on the left) and Character (on the right) cubes and associated profiles. TCI dimensions in profiles: N/n, Novelty Seeking; H/h, Harm Avoidance; R/r, Reward Dependence; S/s, Self-Directedness; C/c, Cooperativeness; T/t, Self-Transcendence.

TCI data are possibly handled in three different ways. Firstly, as total scores on each TCI dimension (numeral dimensions). Secondly, as binary (dichotomized) TCI dimensions (high or low). Lastly, as 8 Temperament and 8 Character profiles formed by all possible combinations of high/low binary TCI dimensions within the Temperament and Character cubes, respectively. For a more convenient depiction of these specific TCI profiles, each TCI dimension is represented by a letter, usually the first letter of its name; a capital letter (N,H,R,P,S,C,T) corresponds to high scores in a dimension, while a lower-case letter (n,h,r,p,s,c,t) refers to low scores. Then, each Temperament or Character profile is represented by the combination of three upper- or lower-case letters. Apart from this three-letter representation, the profiles are dubbed by Cloninger in an additional way, i.e., one that correlates profiles to distinct psychopathologic conditions. The Temperament and Character profiles as proposed by Cloninger are illustrated in **Figure 1** and described in detail in the **Supplementary Material**.

TCI-140 has been translated in Greek and validated in a general population sample (36). Internal consistency was satisfactory, with Cronbach’s alphas ranging between 0.72 and 0.87 across all dimensions: 0.72 (NS), 0.81 (HA), 0.72 (RD), 0.87 (P), 0.80 (SD), 0.84 (CO) and 0.72 (ST). In our sample, we also assessed the internal consistency of each TCI dimension, separately for controls and patients. For controls, Cronbach’s alpha ranged between 0.59 and 0.88 across all dimensions, and the specific values were 0.59 (NS), 0.73 (HA), 0.71 (RD), 0.80 (P), 0.88 (SD), 0.74 (CO), 0.78 (ST). As for patients, it ranged between 0.69 and 0.88, with the particular values being 0.71 (NS), 0.85 (HA), 0.69 (RD), 0.88 (P), 0.87 (SD), 0.82 (CO), 0.85 (ST).

Participants completed the TCI in a single visit without time constraints and no notable distractions in their environment affecting performance. There was a mental health professional present to explain procedures or address questions.

### 2.3 Statistical analysis

All quantitative variables were checked for normality using the Shapiro-Wilk test. Cronbach’s alpha was employed to investigate the internal consistency of the TCI dimensions in both patients and controls. Descriptive data were calculated for all variables across groups (controls, patients with BD, BDI and BDII). Group comparisons were conducted with t-test, Mann-Whitney’s U test or Chi square test, as appropriate. Spearman correlations between TCI dimensions and AAO with potential covariates were performed. To estimate associations of BD diagnosis with TCI dimensions, linear regression modeling was implemented with sex, age, and educational attainment as covariates.

To dichotomize TCI dimensions, population medians were required, which were obtained from Vitoratou et al.’s assessment in a Greek general population sample (n=306) (36) after contact with the author, with permission for use. Furthermore, that sample displayed normally distributed TCI dimension scores, therefore the published means and provided medians were approximately equal. No statistically significant differences were found in sex and age between our BD sample and Vitoratou et al.’s general population sample. Consequently, each TCI dimension in our BD sample was dichotomized utilizing these population medians, with scores below the median classified as “Low”, and scores equal to or above it classified as “High”. However, comparing our control sample with Vitoratou et al.’s population sample revealed a statistically different age composition between the groups, with the former being younger (**Supplementary Table 1**). For this reason, population medians could not be applied to our control sample, therefore their TCI scores were only analyzed as numeral dimensions.

For the first objective, participants were grouped by outcome (i.e., patients with BD, BDI or BDII diagnosis vs. controls) and differences in exposure (i.e., Temperament and Character) were investigated in retrospect (case-control analyses). Logistic regression models assessed the association between numeral TCI dimensions and the probability of BD and subtypes diagnoses, after adjusting for confounders.

For the second objective (case-only analyses), two different regression models were employed, with appropriate adjustment for confounders. Firstly, linear regression models investigated associations of numeral and binary TCI dimensions and individual profiles with age of BD onset. Secondly, Cox regression models (survival analyses) were utilized, using onset of first mood episode as the failure event, AAO as the time variable and numeral and binary TCI dimensions and individual profiles as predictors. Survival Kaplan-Meier curves were produced to depict the TCI profiles associated with the latest (most protective) and earliest (most harmful) disease onset.

In summary, numeral TCI dimensions were used in all regression models (logistic, linear, and Cox), while binary dimensions and TCI profiles were applied only in linear and Cox regressions. In all analyses, each numeral or binary TCI dimension was always analyzed separately from the other dimensions.

To select confounders of the exposure-outcome association in our multivariate models, we prioritized variables associated with the exposure (TCI dimensions), causally associated with the outcome (BD diagnosis in the total sample or BD AAO in the patient sample) or both, predating outcome and preferably exposure and not being mediators in the etiological pathway between exposure and outcome, adopting a causal perspective (37). Sex, age (proxy for birthdate), educational attainment, and psychiatric family history were all assumed to predate outcome. Family status or employment were *a priori* excluded as potential confounders as they were recorded at recruitment, i.e., following the outcome (BD onset). Even in cases where these variables also reflected pre-onset status, they would be at best considered as mediators in the pathway from exposure (TCI traits) to outcome (BD diagnosis or onset).

To analyze the 8 profiles of Temperament or Character (representing all possible combinations of “Low” and “High” scores in the binary dimensions of each cube), two nominal variables with 8 levels each were created and inserted as independent variables in the linear and Cox regressions. We used the STATA command “Margins” to obtain predicted B coefficients with linear regressions or linear predictions (log ORs/HRs) with logistic and Cox regressions, respectively, for each profile. “Margins”, therefore, pinpointed the most extreme Temperament and Character profiles (highest or lowest predicted estimates). Using these extremes as base in the regressions, profiles significantly different from the most harmful one were considered protective, and those significantly differing from the most protective profile were considered harmful.

To reduce experiment-wise error from multiple comparisons across the 8-level Temperament and Character profile variables, two levels of Bonferroni correction were applied. First, a modified, more liberal correction was used, lowering the significance threshold from 5% to 2.5% (p < 0.025), due to each analysis being run twice (once using the most harmful and once the most protective profile as base). Then, a stricter typical Bonferroni correction (p < 0.0018) was applied to account for all 28 pairwise comparisons within the 8-profile Temperament and Character variables. The initial exploratory liberal approach was utilized to detect potentially relevant findings that might otherwise be obscured by small group sizes.

All analyses were carried out using STATA 13.0 statistical software.

## 3 Results

### 3.1 Descriptive statistics

None of the outpatients approached rejected participation. Initially, 280 participants were included (181 BD patients, 99 healthy controls) but 5 TCI inventories (2 patients’ and 3 healthy controls’) were invalid due to missingness (>10% statements of any TCI dimension unanswered). Therefore, the final sample consisted of 275 individuals, i.e. 179 BD patients (112 BDI, 67 BDII) and 96 healthy controls, yielding a patient-to-control ratio of almost 2:1. In the final sample, all questionnaires were complete and valid as all four TCI validity items were returned correct by the participants.

Mean age of patients and controls was 47.99 years (SD = 12.01) and 40.36 years (SD = 12.61), respectively. The sample included predominantly females (62.57% for patients and 63.54% for controls). Apart from sex, there was a statistically significant difference in all other sociodemographic variables investigated across the two groups (age, educational attainment, employment status, family status). More specifically, the patient sample presented with higher age, lower educational attainment, higher rates of unemployment and retirement, as well as higher rates of divorce/separation (**Table 1**).

**Table 1:** Socio-demographic characteristics and Temperament and Character Inventory (TCI) dimensions for patients with BD and controls.

Variable	Controls (N = 96)	BD (N = 179)	BDI (N = 112)	BDII (N = 67)	BD vs. Controls	BDI vs. Controls	BDII vs. Controls
Age ^a^	40.36 (12.61)	47.99 (12.01)	45.80 (12.36)	51.64 (10.51)	**p<0.001**	**p=0.002**	**p<0.001**
Male Sex ^b^	35 (36.46%)	67 (37.43%)	47 (41.96%)	20 (29.85%)	p=0.874	p=0.418	p=0.380
Employment Status^b^					**p<0.001**	**p<0.001**	**p<0.001**
Unemployed/Students	24 (25.00%)	68 (37.99%)	45 (40.18%)	23 (34.33%)			
Employed	68 (70.83%)	66 (36.87%)	41 (36.61%)	25 (37.31%)			
Retired	4 (4.17%)	45 (25.14%)	26 (23.21%)	19 (28.36%)			
Family Status^b^					**p<0.001**	**p<0.001**	**p<0.001**
Single	36 (37.50%)	53 (29.61%)	40 (35.71%)	13 (19.40%)			
Married/Cohabiting	57 (59.38%)	69 (38.55%)	40 (35.71%)	29 (43.28%)			
Separated/Widowed	3 (3.13%)	57 (31.84%)	32 (28.57%)	25 (37.31%)			
Educational attainment (years) ^c^	16 (13-18)	14 (12-16)	14 (12-16)	14 (12-16)	**p<0.001**	**p<0.001**	**p=0.002**
Novelty Seeking ^d^	53.43 (7.72)	60.85 (10.17)	60.71 (9.76)	61.07 (10.90)	**8.23** **p<0.001** **[5.78, 10.69]**	**7.94** **p<0.001** **[5.41, 10.46]**	**9.07** **p<0.001** **[5.87, 12.27]**
Harm Avoidance ^d^	54.40 (9.21)	62.91 (13.82)	59.83 (12.36)	68.04 (14.67)	**8.36** **p<0.001** **[5.15, 11.57]**	**5.63** **p=0.001** **[2.48, 8.78]**	**13.64** **p<0.001** **[9.60, 17.67]**
Reward Dependence ^d^	69.08 (8.98)	67.38 (9.78)	67.55 (9.86)	67.09 (9.71)	-1.81 p=0.145 [-4.24, 0.63]	-1.59 p=0.236 [-4.21, 1.04]	-1.89 p=0.244 [-5.07, 1.30]
Persistence ^d^	70.50 (9.42)	67.00 (13.98)	68.90 (13.37)	63.82 (14.50)	-2.65 p=0.113 [-5.93, 0.63]	-1.16 p=0.495 [-4.49, 2.18]	**-6.17** **p=0.004** **[-10.29, -2.05]**
Self-Directedness ^d^	80.15 (11.27)	64.27 (14.61)	67.03 (14.21)	59.66 (14.20)	**-14.54** **p<0.001** **[-18.08, -11.0]**	**-12.01** **p<0.001** **[-15.70, -8.32]**	**-20.02** **p<0.001** **[-24.44, -15.6]**
Cooperativeness ^d^	79.05 (8.41)	74.35 (11.99)	74.40 (11.71)	74.27 (12.54)	**-4.56** **p=0.002** **[-7.39, -1.73]**	**-4.56** **p=0.002** **[-7.49, -1.63]**	**-5.36** **p=0.004** **[-8.94, -1.78]**
Self-Transcendence ^d^	38.70 (8.93)	44.56 (12.70)	46.00 (12.88)	42.16 (12.11)	**5.30** **p=0.001** **[2.26, 8.33]**	**6.77** **p<0.001** **[3.54, 10.00]**	2.15 p=0.245 [-1.49, 5.79]
Age of Onset (years)	–	31.56 (11.78)	29.29 (10.76)	35.37 (12.48)	–	–	–

In the first 4 columns, Mean (SD) or Median (25^th^ – 75^th^ percentile) or N (%) are presented for sociodemographic characteristics and TCI dimensions.

In the last 3 columns, the following are presented: ^a^ t-test, ^b^ Chi square test, ^c^ Mann-Whitney’s U-test, ^d^ the effect of BD diagnosis (B, p-value and [95% CIs]) in linear regressions of TCI dimensions on BD diagnosis after adjusting for age, sex and educational attainment. Bold, p<0.05.

Total BD patients’ mean AAO was 31.56 years (SD = 11.78), while for BDI patients particularly it was 29.29 years (SD = 10.76) and for BDII patients it was 35.37 years (SD = 12.48). Median number of depressive, manic and hypomanic episodes was 3, 1 and 1, respectively. Median number of hospitalizations was 2. More than half (53.1%) had a lifetime history of psychotic symptoms while 46.4% had attempted suicide. Almost two-thirds had a depressive onset polarity (65.4%). Predominant polarity was depressive in 38.6% and hyperthymic in 24%. Almost half (49.2%) had a positive family history of major psychiatric disorder without significant differences among BD subgroups (BDI 48.2%, BDII 50.8%).

Age, as well as all TCI dimensions apart from RD and CO followed a normal distribution for controls and patients alike, while RD and CO showed normality for patients exclusively. However, adhering to the central limit theorem, normality was assumed for these two variables as well. Vitoratou’s assessment in the general Greek population reinforced this assumption; that sample followed the normal distribution on all TCI dimensions, as well. AAO, number of episodes and hospitalizations were not normally distributed.

Spearman correlations of TCI dimensions with potential covariates in the total sample showed that female sex was associated with higher HA (ρ=0.18, p=0.002), RD (ρ=0.23, p<0.001) and CO (ρ=0.19, p=0.002), higher age was associated with lower SD (ρ=-0.26, p<0.001), lower P (ρ=-0.13, p=0.03) and higher ST (ρ=0.15, p=0.01), while education was positively associated only with SD (ρ=0.27, p<0.001).

Initial assessment with linear regressions for effects of BD diagnosis on TCI numeral dimensions after adjusting for age, sex and educational attainment are also demonstrated in **Table 1**. All in all, the NS, HA, and ST dimensions displayed a positive association with BD (with the exception of BDII not significantly associated with ST), while SD and CO were negatively associated with the disease. RD and P provided no statistically significant associations, with the exception of BDII being negatively associated with the P dimension. **Table 2** presents absolute and relative frequencies of each Temperament and Character profile in the patient sample, as well as means (SD) for AAO per TCI profile.

**Table 2:** Distribution of cases (BD patient group) and age of disease onset by Temperament and Character profiles.

TCI profile	BD PATIENTS (N = 179)	
	N (%)	Age of onset: Mean (SD)
Temperament profile		
nhr/Schizoid	8 (4.47)	32.8 (20.0)
Nhr/Antisocial	14 (7.82)	32.1 (11.3)
nHr/Obsessive	15 (8.38)	32.9 (10.4)
nhR/Reliable	12 (6.70)	38.3 (16.2)
NHr/Borderline	30 (16.76)	31.7 (12.3)
NhR/Histrionic	28 (15.64)	29.6 (9.2)
nHR/Cautious	25 (13.97)	35.1 (12.0)
NHR/Narcissistic	47 (26.26)	28.3 (9.6)
Character Profile		
sct/Melancholic	20 (11.17)	28.8 (10.7)
Sct/Bossy	18 (10.06)	37.0 (12.6)
sCt/Dependent	15 (8.38)	31.9 (8.4)
scT/Schizotypal	17 (9.50)	29.8 (10.9)
SCt/Organized	45 (25.14)	32.1 (12.6)
ScT/Paranoid	13 (7.26)	29.4 (13.2)
sCT/Cyclothymic	23 (12.85)	29.5 (9.7)
SCT/Creative	28 (15.64)	32.7 (13.4)

TCI dimensions in profiles: N/n, Novelty Seeking; H/h, Harm Avoidance; R/r, Reward Dependence; S/s, Self-Directedness; C/c, Cooperativeness; T/t, Self-Transcendence

### 3.2 Case-control analyses (BD risk prediction)

The logistic regression analyses on numeral TCI dimensions are displayed in **Table 3**. Based on associations of TCI dimensions and BD diagnosis (outcome) with potential covariates, age, sex, and educational attainment were selected as confounders for these analyses. Family history had no variability among controls and was therefore not included as a confounder for this part of the study. Risk of BD was significantly predicted by higher NS, HA, ST and lower SD and CO. When comparing BDI patients specifically versus controls, the results closely mirrored the overall findings for BD. Results were more varied for BDII patients. NS and HA but not ST showed a significant positive association with BDII versus controls while SD and CO as well as P had a significant negative association with BDII risk.

**Table 3:** Logistic regressions of BD and subtypes (BDI, BDII) diagnosis on numeral Temperament and Character Inventory (TCI) dimensions.

TCI dimension	BD vs. Controls	BDI vs. Controls	BDII vs. Controls
NS	**1.11** **p<0.001** **[1.07, 1.15]**	**1.12** **p<0.001** **[1.07, 1.17]**	**1.12** **p<0.001** **[1.07, 1.17]**
HA	**1.06** **p<0.001** **[1.03, 1.09]**	**1.05** **p=0.001** **[1.02, 1.08]**	**1.11** **p<0.001** **[1.07, 1.16]**
RD	0.98 p=0.164 [0.95, 1.01]	0.98 p=0.249 [0.95, 1.01]	0.98 p=0.262 [0.94, 1.02]
P	0.98 p=0.122 [0.96, 1.00]	0.99 p=0.521 [0.97, 1.01]	**0.96** **p=0.005** **[0.93, 0.99]**
SD	**0.92** **p<0.001** **[0.90, 0.94]**	**0.93** **p<0.001** **[0.90, 0.95]**	**0.90** **p<0.001** **[0.87, 0.93]**
CO	**0.96** **p=0.002** **[0.93, 0.98]**	**0.96** **p=0.003** **[0.93, 0.99]**	**0.95** **p=0.006** **[0.91, 0.98]**
ST	**1.04** **p=0.001** **[1.02, 1.07]**	**1.06** **p<0.001** **[1.03, 1.09]**	1.02 p=0.245 [0.99, 1.05]

Odds Ratios, p-values [95% CIs] in logistic regressions of BD diagnosis on each numeral TCI dimension after adjusting for age, sex and educational attainment.

TCI dimensions abbreviations: NS, Novelty Seeking; HA, Harm Avoidance; RD, Reward Dependence; P, Persistence; SD, Self-Directedness; CO, Cooperativeness; ST, Self-Transcendence Bold, p<0.05.

### 3.3 Case-only analyses (age of onset prediction)

The analyses for the second objective (linear and Cox regressions) were performed in patients only. Age of BD onset was significantly positively correlated with BDII diagnosis (vs. BDI, ρ=0.25, p<0.001), age (ρ=0.50, p<0.0001) and negatively correlated with family history of major psychiatric disorder (ρ=-0.18, p=0.016) but not sex and educational attainment. Spearman correlations of TCI dimensions with potential covariates in the patient sample showed that female sex was associated with higher HA (ρ=0.24, p=0.001), RD (ρ=0.21, p=0.005) and CO (ρ=0.17, p=0.03), education was positively associated only with SD (ρ=0.21, p=0.005), BDII subtype (vs. BDI) was associated with higher HA (ρ=0.28, p<0.001), lower P (ρ=-0,18, p=0.016) and lower SD (ρ=-0.26, p<0.001), while age and family history of major psychiatric disorder showed only non-significant associations with TCI dimensions.

Based on associations of TCI dimensions and BD AAO (outcome) with potential covariates (age, sex, educational attainment, family history of major psychiatric disorder and BD subtype), sex, educational attainment and family history were selected as confounders for both case-only regression models. Family history was selected as it presented non-zero variability within patients and was negatively correlated with onset age. Age was not significantly correlated with TCI dimensions in the patient sample (despite having significant associations in the overall sample) but was significantly positively correlated with age of BD onset. The latter was mainly considered a nuisance ‘non-causal’ effect of recruitment; patients’ AAO had to be up to their age, i.e., it was censored by age, or, in other words, young patients would necessarily have earlier onset. Therefore, age was excluded from patient-only analyses. Finally, BD subtype was also included as confounder only in the analyses in the whole BD sample, to adjust for its BDI/BDII composition. Additionally, subgroup analyses by BDI/BDII subtype were implemented.

#### 3.3.1 Numeral and binary TCI dimensions

**Table 4** includes linear regression analyses among patients with BD and subtypes with AAO as the dependent variable and binary TCI dimensions as predictors, adjusting for BD subtype (only in the whole BD sample), sex, educational attainment, and family history of major psychiatric disorder. NS and SD appeared to be the only dimensions attaining statistical significance in the total BD sample. More specifically, patients with BD with NS scores higher than the population’s median had earlier AAO compared to those with a lower than median score. The opposite held true for SD. High scorers for SD appeared to have a delayed onset of BD. These findings also held for BDI patients but were not supported for BDII patients. High ST and high P appeared to be significantly associated with earlier onset of disease in BDI patients only. Linear regressions of AAO on the numeral TCI dimensions, also in **Table 4**, held for the most part similar, if slightly less pronounced findings than the binary dimensions. While numeral NS retained its significance in BD and BDI patients, the numeral SD dimension was not statistically significant across BD and subtypes, as opposed to the binary variable. For BDI patients, numeral ST preserved its significance while the P dimension became non-significant when analyzed as numeral.

**Table 4:** Linear regressions of age of disease onset among patients with BD and subtypes (BDI, BDII) on numeral and binary Temperament and Character Inventory (TCI) dimensions.

TCI dimension	BD (N = 179)		BDI (N = 112)		BDII (N = 67)	
	Numeral	Binary	Numeral	Binary	Numeral	Binary
NS	**-0.24** **p=0.003** **[-0.40, -0.08]**	**-4.70** **p=0.007** **[-8.10, -1.30]**	**-0.21** **p=0.031** **[-0.41, -0.02]**	**-4.44** **p=0.029** **[-8.41, -0.47]**	-0.27 p=0.058 [-0.56, 0.01]	-5.08 p=0.125 [-11.62, 1.45]
HA	-0.06 p=0.332 [-0.19, 0.06]	-1.28 p=0.480 [-4.84, 2.29]	-0.05 p=0.576 [-0.20, 0.11]	-1.34 p=0.507 [-5.34, 2.65]	-0.09 p=0.423 [-0.31, 0.13]	-1.25 p=0.752 [-9.16, 6.66]
RD	-0.08 p=0.354 [-0.25, 0.09]	-0.95 p=0.585 [-4.36, 2.47]	0.03 p=0.741 [-0.17, 0.24]	-0.18 p=0.932 [-4.31, 3.96]	-0.28 p=0.085 [-0.60, 0.04]	-2.48 p=0.447 [-8.97, 4.01]
P	-0.06 p=0.346 [-0.18, 0.06]	-0.60 p=0.722 [-3.95, 2.74]	-0.12 p=0.110 [-0.26, 0.03]	**-4.25** **p=0.034** **[-8.18, -0.32]**	0.04 p=0.740 [-0.18, 0.26]	5.46 p=0.079 [-0.66, 11.59]
SD	0.10 p=0.090 [-0.02, 0.22]	**5.24** **p=0.003** **[1.87, 8.62]**	0.10 p=0.151 [-0.04, 0.24]	**4.73** **p=0.023** **[0.66, 8.80]**	0.11 p=0.339 [-0.11, 0.33]	5.93 p=0.059 [-0.24, 12.09]
CO	0.02 p=0.825 [-0.12, 0.15]	0.53 p=0.759 [-2.86, 3.91]	0.07 p=0.419 [-0.10, 0.24]	1.08 p=0.593 [-2.91, 5.07]	-0.08 p=0.561 [-0.33, 0.18]	-0.63 p=0.851 [-7.35, 6.08]
ST	-0.02 p=0.748 [-0.15, 0.11]	-1.23 p=0.463 [-4.52, 2.07]	**-0.15** **p=0.047** **[-0.30, 0.00]**	**-3.99** **p=0.039** **[-7.79, -0.20]**	0.23 p=0.073 [-0.02, 0.48]	3.67 p=0.242 [-2.55, 9.89]

Derived B coefficients, p-values [95% CIs] from linear regressions of age of onset on each TCI dimension after adjusting for BD subtype (only in the total BD sample), sex, educational attainment, and family history of major psychiatric disorder. Analyses with binary dimensions compare high vs. low scores in each dimension.

TCI dimensions abbreviations: NS, Novelty Seeking; HA, Harm Avoidance; RD, Reward Dependence; P, Persistence; SD, Self-Directedness; CO, Cooperativeness; ST, Self-Transcendence Bold, p<0.05.

**Figure 2:**
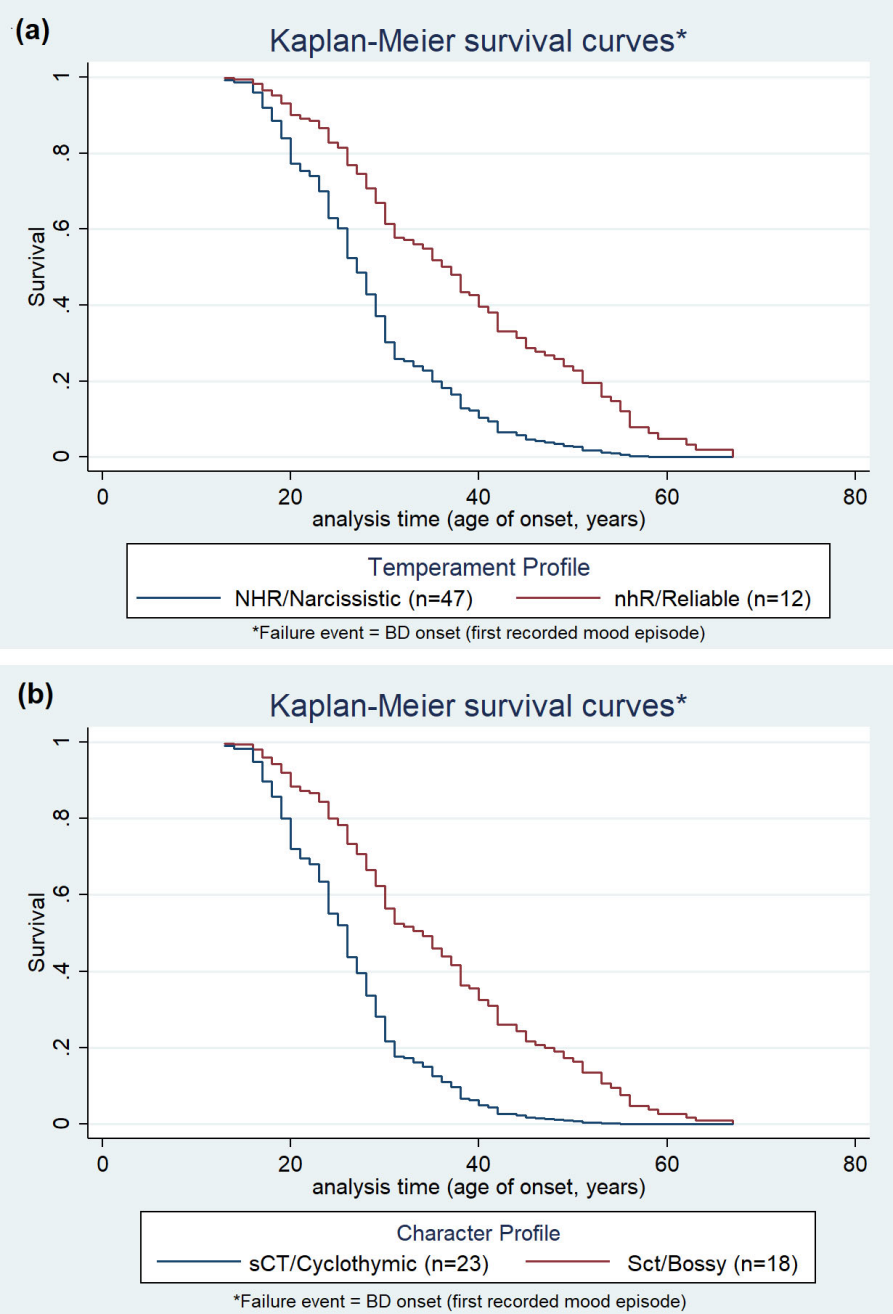
Survival curves of the Temperament **(a)** and Character **(b)** profiles associated with the earliest (most ‘harmful’, blue color) and the latest (most ‘protective’, red color) BD onset in the patient sample. TCI dimensions in profiles: N/n, Novelty Seeking; H/h, Harm Avoidance; R/r, Reward Dependence; S/s, Self-Directedness; C/c, Cooperativeness; T/t, Self-Transcendence Mean (SD) BD age of onset (y) per profile: **(a)** NHR/Narcissistic: 28.3 (9.6), nhR/Reliable: 38.3 (16.2), **(b)** sCT/Cyclothymic: 29.5 (9.7), Sct/Bossy: 37.0 (12.6).

**Table 5** demonstrates the results of the Cox regressions in the total BD sample and subtypes, with AAO as the time variable and the binary TCI dimensions as predictors, adjusting for BD subtype (only in the whole BD sample), sex, educational attainment and family history of major psychiatric disorder. As analyses were performed in patients only, all observations eventually showcase the failure event, as presented in the Kaplan-Meier curves (**Figures 2a, b**). Following the trend marked by previous models, high NS scores displayed an increased hazard ratio for BD onset by 60%, and inversely high SD scores showed a reduced risk of BD onset by around 40%, suggesting that high NS and high SD were associated with earlier and later BD onset, respectively, than low NS and low SD. These findings remained significant across subtypes except for NS in BDII patients. High P corresponded to a significantly higher risk of onset by about 50% exclusively in BDI. Cox regressions on the numeral TCI dimensions are also displayed in **Table 5**. Following the trend described in linear regressions, the SD numeral dimension lost its statistical significance across all BD groups (BD total, BDI and BDII). Numeral NS, on the other hand, retained its statistical significance in BD and BDI patients. Furthermore, numeral NS was also associated with earlier onset in BDII patients unlike its binary counterpart. Finally, P lost its significance in BDI patients when analyzed as numeral.

**Table 5:** Cox regressions of disease onset on numeral and binary Temperament and Character Inventory (TCI) dimensions among patients with BD and subtypes (BDI, BDII).

TCI dimension	BD (N = 179)		BDI (N = 112)		BDII (N = 67)	
	Numeral	Binary	Numeral	Binary	Numeral	Binary
NS	**1.03** **p=0.001** **[1.01, 1.04]**	**1.60** **p=0.005** **[1.15, 2.22]**	**1.02** **p=0.031** **[1.00, 1.04]**	**1.58** **p=0.033** **[1.04, 2.42]**	**1.03** **p=0.026** **[1.00, 1.06]**	1.53 p=0.129 [0.88, 2.64]
HA	1.00 p=0.464 [0.99, 1.02]	1.13 p=0.452 [0.82, 1.58]	1.00 p=0.744 [0.99, 1.02]	1.11 p=0.616 [0.74, 1.65]	1.01 p=0.540 [0.99, 1.03]	1.13 p=0.715 [0.59, 2.14]
RD	1.01 p=0.417 [0.99, 1.02]	1.11 p=0.522 [0.81, 1.51]	1.00 p=0.894 [0.98, 1.02]	1.05 p=0.803 [0.70, 1.58]	1.03 p=0.072 [1.00, 1.05]	1.28 p=0.393 [0.72, 2.28]
P	1.01 p=0.248 [1.00, 1.02]	1.16 p=0.349 [0.85, 1.58]	1.01 p=0.108 [1.00, 1.02]	**1.52** **p=0.042** **[1.02, 2.26]**	1.00 p=0.895 [0.98, 1.02]	0.69 p=0.161 [0.41, 1.16]
SD	0.99 p=0.076 [0.98, 1.00]	**0.57** **p=0.001** **[0.40, 0.79]**	0.99 p=0.168 [0.98, 1.00]	**0.60** **p=0.019** **[0.39, 0.92]**	0.99 p=0.343 [0.97, 1.01]	**0.55** **p=0.034** **[0.32, 0.96]**
CO	1.00 p=0.786 [0.99, 1.01]	0.97 p=0.848 [0.71, 1.32]	0.99 p=0.457 [0.98, 1.01]	0.91 p=0.640 [0.62, 1.34]	1.01 p=0.488 [0.99, 1.03]	1.16 p=0.610 [0.66, 2.05]
ST	1.01 p=0.410 [0.99, 1.02]	1.16 p=0.318 [0.86, 1.57]	1.01 p=0.068 [1.00, 1.03]	1.39 p=0.088 [0.95, 2.04]	0.99 p=0.182 [0.96, 1.01]	0.83 p=0.461 [0.51, 1.36]

Hazard ratios, p-values [95% CIs] in Cox regressions of BD onset on each TCI dimension after adjusting for BD subtype (only in the total BD sample), sex, educational attainment, and family history of major psychiatric disorder. Analyses with binary dimensions compare high vs. low scores in each dimension.

TCI dimensions abbreviations: NS, Novelty Seeking; HA, Harm Avoidance; RD, Reward Dependence; P, Persistence; SD, Self-Directedness; CO, Cooperativeness; ST, Self-Transcendence Bold, p<0.05.

#### 3.3.2 TCI profile analyses

Linear and Cox regressions were then applied to specific Temperament and Character profiles of the patient sample, after adjusting for BD subtype (only in the whole BD sample), sex, educational attainment, and family history of major psychiatric disorder. To account for the number of comparisons, two levels of Bonferroni correction were applied, first a more liberal approach followed by a stricter, conservative Bonferroni correction. The application of the conservative approach produced no statistically significant results. The results after applying the more liberal version of Bonferroni are presented in **Table 6**. In both linear and Cox (**Figure 2a**) regressions, the Temperament profiles most associated with the latest (protective) and earliest onset (harmful) were nhR (Reliable) and NHR (Narcissistic), respectively. NhR (Histrionic) was also harmful, significant in linear regressions and nominally significant in Cox regressions. NHr (Borderline) was also harmful but only nominally significant in linear regressions. nHR (Avoidant) was protective but only nominally significant in Cox regressions. For BDI in particular, nhR (Reliable) appeared to be a profile associated with later onset significantly in linear regressions and nominally significantly in Cox regressions, while the NHR (Narcissistic), nhr (Schizoid), NHr (Borderline) and NhR (Histrionic) profiles appeared to be correlated with earlier onset in linear regressions (from strongest to weakest correlation, with only NHR (Narcissistic) yielding significant results). Cox regressions for BDI yielded only nominally significant results for the NHR (Narcissistic) profile, while all other profiles (nhr, NHr, NhR) lost their significance. For BDII in particular, nhr (Schizoid) appeared to be the profile associated with latest onset in linear regressions (only nominally significantly in Cox regressions). The profiles associated with earlier onset, from strongest to weakest associations, were NHR (Narcissistic), Nhr (Antisocial), NhR (Histrionic), and the nominally significant nHr (Obsessional), NHr (Borderline) and nHR (Avoidant). These associations were weakened in Cox regressions, and only the NHR (Narcissistic) and NhR (Histrionic) profiles remained as nominally significant, while all others were rendered non-significant.

**Table 6:** Results of the 8 Temperament and 8 Character profile analyses among patients with BD and subtypes (BDI, BDII) regarding disease onset.

Diagnosis	Analysis method	Protective		Harmful	
		Temperament	Character	Temperament	Character
BD (N = 179)	Linear^1^	**nhR/Reliable**	**Sct/Bossy** **SCT/Creative** SCt/Organized	**NHR/Narcissistic NhR/Histrionic** NHr/Borderline	**sct/Melancholic sCT/Cyclothymic**
	Survival^2^	**nhR/Reliable** nHR/Avoidant	**Sct/Bossy** **SCt/Organized** **SCT/Creative**	**NHR/Narcissistic** NhR/Histrionic	**sCT/Cyclothymic** **sct/Melancholic**
BDI (N = 112)	Linear^1^	**nhR/Reliable**	**Sct/Bossy SCt/Organized**	**NHR/Narcissistic** nhr/Schizoid NHr/Borderline NhR/Histrionic	**sCT/Cyclothymic**
	Survival^2^	nhR/Reliable	**Sct/Bossy SCt/Organized** SCT/Creative	NHR/Narcissistic	**sCT/Cyclothymic**
BDII (N = 67)	Linear^1^	**nhr/Schizoid**	**Sct/Bossy** **SCT/Creative**	**NHR/Narcissistic Nhr/Antisocial NhR/Histrionic** nHr/Obsessional NHr/Borderline nHR/Avoidant	**sct/Melancholic** sCt/Dependent SCt/Organized
	Survival^2^	nhr/Schizoid	Sct/Bossy SCT/Creative	NHR/Narcissistic NhR/Histrionic	sCt/Dependent

^1^ Linear regressions of BD, BDI or BDII age of onset on TCI profiles after adjusting for BD subtype (only in the total BD sample), sex, educational attainment, and family history of major psychiatric disorder. Harmful is indicated by Β-coefficient<0, and protective by B-coefficient>0.

^2^ Cox regressions of BD, BDI or BDII onset on TCI profiles after adjusting for BD subtype (only in the total BD sample), sex, educational attainment, and family history of major psychiatric disorder. Harmful is indicated by HR>1 and protective by HR<1.

When applying liberal Bonferroni correction (p-value divided by 2), profiles with statistically significant p-values after *post-hoc* correction are bolded (p<0.025), and nominally significant profiles are displayed unbolded (0.025 < p < 0.05); profiles failing to reach at least nominal significance are not displayed. When applying conservative Bonferroni corrections taking into account all 28 pairwise comparisons between profiles, no profile remained statistically significant.

Profiles are sorted from strongest to weakest in terms of the significance of their protective or harmful effects.

TCI dimensions in profiles: N/n, Novelty Seeking; H/h, Harm Avoidance; R/r, Reward Dependence; S/s, Self-Directedness; C/c, Cooperativeness; T/t, Self-Transcendence

With the liberal application of Bonferroni on Character profiles, Sct (Bossy) was the profile most associated with later disease onset in linear and Cox regressions, followed by SCT (Creative) and SCt (Organized), which was only nominally significant in linear regressions, while sCT (Cyclothymic) and sct (Melancholic) were associated with earlier onset (the most extreme profiles are showcased in **Figure 2b**). For BDI in particular, two profiles yielded statistically significant associations for later onset both in linear and Cox regressions; Sct (Bossy) followed by SCt (Organized), with SCT (Creative) as a third nominally significant protective profile being found exclusively in Cox regressions. Only one profile, sCT (Cyclothymic), was significantly associated with earlier onset for BDI, both in linear and Cox regressions. In the case of BDII, two profiles significantly associated with later onset in linear regressions were Sct (Bossy) followed by SCT (Creative), and both retained only nominal significance in Cox regressions. The only significant profile associated with earlier onset for BDII in linear regressions was sct (Melancholic) followed by the nominally significant sCt (Dependent) and SCt (Organized), while in Cox regressions only sCt (Dependent) remained nominally significant and the other profiles lost their relevance.

## 4 Discussion

Regarding the first objective about associations between Temperament and Character and risk of BD, two Temperament and two Character numeral dimensions appeared to bear strong associations across BD and subtypes; high NS and HA were associated with higher BD risk while high SD and CO with lower BD risk. BD subtype-specific effects involved high ST increasing risk of BDI and high P decreasing risk of BDII. As this study’s control sample and the general population validation sample differed on age, it would not be valid to apply population medians to our control sample to binarize TCI dimensions and create profiles. Using the control sample’s own medians and applying the population medians only to patients, i.e., using two separate sets of medians for controls and patients in a single model, would also be completely invalid as exposure (TCI binary dimensions or profiles) would be defined differently in the two samples. Therefore, comparisons between patients and controls could not be extended to TCI binary dimensions and profiles, and the first objective was not fully explored, rendering it a topic that warrants further illumination in future research.

As for the second objective about associations between Temperament and Character and BD AAO, NS appeared to be the prevailing numeral and binary dimension associated with earlier onset, while conversely SD seemed to relate to delayed onset as a binary dimension. These associations were less prominent in BDII while in BDI earlier onset was also associated with high ST (numeral and binary) and high binary P. Finally, profile analyses among patients yielded several interesting results.

We chose to analyze each numeral or binary TCI dimension separately from the others rather than in multivariate models including all TCI dimensions together. Our choice was justified by the fact that correlations between dimensions were allowed during the construction of the TCI inventory and have indeed been recorded especially within the Character Cube (between SD and CO) but also across cubes (e.g. between RD and CO, and between HA and SD) (25). In contrast, correlations are minimal within the Temperament Cube, whose dimensions are more independent and genetically homogenous (38, 39). Therefore, intercorrelations between dimensions both within and across cubes, potentially masking some true effects, were avoided by separately analyzing each dimension.

Regarding the internal consistency of the TCI dimensions, it was generally acceptable (α > 0.7), with higher values attributed to the patient sample, which followed the α values from Vitoratou et al.’s general population sample more closely than the control sample. Only two values were lower than the cutoff; NS for controls (α = 0.59) and RD for patients (α = 0.69). These dimensions have also shown low alpha values in other studies (36, 40, 41). The low internal consistency for NS in controls and borderline value for RD in patients warrants caution interpreting results involving these dimensions, as well as associated Temperament profiles in the case of RD. It could indicate heterogeneity in the items assessed within a scale, poor wording or understanding by the subjects, or a complexity of the construct being assessed. In any case, low internal consistency might reflect higher measurement error, lower precision, and any definitive statements involving these dimensions would be wise to be withheld.

Several studies have linked BD with personality characteristics from various other theoretical models. The FFM model (21, 42) relates BD mainly to high neuroticism but investigation of other dimensions has produced mixed results; a recent meta-analysis found higher neuroticism and lower conscientiousness and extraversion in patients with BD compared to controls, with large, medium and small effect sizes, respectively (43). Another recent study utilizing the Swedish universities Scales of Personality (SSP) associates BD with aspects of Neuroticism, Aggressiveness and Disinhibition (44). Finally, a review of 81 articles links BD with distinct positive personality traits, such as spirituality, empathy, creativity, realism and resilience (45).

The NS and HA numeral dimensions related to increased risk of BD across samples compared to controls, and conversely SD and CO were associated with reduced risk across samples, findings that align with established literature (29, 30). ST was also associated with higher risk of BD and BDI also in line with previous literature while the numeral P dimension demonstrated a protective role exclusively towards BDII.

To the author’s knowledge, this is the first study that found significant associations of TCI dimensions in regard to BD and subtypes’ AAO, while the only other relevant study did not merit significant findings, possibly due to sample heterogeneity and differing covariates and methodology (32). Namely, we additionally adjusted for educational attainment and family history but excluded age from our analyses while they also adjusted for inpatient setting. BD subtype was included in both studies, but we also performed subgroup analyses stratified by BD subtype. In our study, high NS and low SD were consistently associated with earlier onset. Therefore, out of all dimensions associated with BD risk, only few (NS, SD) seem to also affect disease onset significantly and in the same direction, indicating a cumulative harmful or protective risk, respectively. Furthermore, high ST was associated with earlier onset exclusively in BDI, in line with the case-control analyses. Interestingly, high P was also linked to earlier onset in BDI, although in case-control analyses it was not associated with BDI risk but with lower BDII risk. This finding suggests opposite direction links of the P dimension with BDI and BDII.

In linear and Cox regressions three distinct patterns of association emerged between TCI numeral and binary dimensions and BD onset. Firstly, some dimensions were associated with BD onset both as numeral and binary variables (e.g., NS with earlier BD/BDI onset, ST with earlier BDI onset), suggesting a strong linear correlation. Secondly, certain dimensions showed significant associations as numeral but not as binary variables (e.g., NS with earlier onset in BDII Cox regressions), indicating a weak linear correlation with a more gradual change in risk of BD onset not captured in the binary dimension. Thirdly, some associations appeared exclusively in binary dimensions (e.g., SD with delayed BD/BDI/BDII onset, P with earlier BDI onset), indicating non-linear correlations between the dimensions and onset. These considerations justify the scope of jointly studying numeral and binary dimensions.

As proposed by Cloninger (27), investigations should not be limited to numeral or binary TCI dimensions, but an even better insight is offered by considering TCI profiles, i.e., the interactions between the binary TCI dimensions of the Temperament or Character Cubes. For example, while all profiles with high NS would be expected to be associated with earlier BD onset, only the NHR (Narcissistic) and NhR (Histrionic) profiles presented a significant association in the total BD sample (applying a liberal Bonferroni correction). This could indicate a key advantage of TCI profiles versus binary dimensions; profiles capture complex interactions of all three dimensions within the cube, offering a more nuanced understanding than dimensions in isolation, even though these analyses lack the power to support any definitive statement (as no profile survived strict Bonferroni correction across all samples). This study was the first attempt to utilize TCI profiles in association with BD onset.

Although profile analyses should be considered as exploratory and did not survive strict Bonferroni correction, some findings are potentially interesting and warrant further investigation in the future. Out of the Temperament profiles, the nhR (Reliable) profile was the most protective (associated with delayed onset), followed by nHR (Avoidant) while NHR (Narcissistic) was the most harmful (earlier onset), followed by NhR (Histrionic) and NHr (Borderline). The NHR (Narcissistic) Temperament profile which reflects traits of Narcissistic PD, was consistently associated with earlier BD onset across samples (more strongly in linear than in Cox regressions). Narcissistic traits often present with emotional extremes; grandiosity followed by intense shame or rage after disappointment (46). Additionally, supporting our findings, individuals with Narcissistic PD display a lifetime comorbidity risk of BDI at 20.1% (47). Similarly, other Temperament profiles associated with cluster B PDs (NhR-Histrionic, NHr-Borderline, Nhr-Antisocial) were also associated with earlier BD, BDI or BDII onset in some analyses, indicating a harmful role, which aligns with established literature; cluster B PDs were included by Kraepelin in the same affective continuum as BD (48) and are more prevalent in patients with BD than the general population (49). Additionally, according to a recent systematic review, comorbid Antisocial PD is associated with earlier BD onset and greater severity (50).

The nHR (Avoidant) and nhr (Schizoid) Temperament profiles were protective regarding disease onset in BD and BDII, respectively. It is important to distinguish between profiles bearing a protective role particularly against BD onset and being generally safe. These profiles are associated with specific PDs (Avoidant and Schizoid PD, respectively), which entail severe impairment in social functioning. Schizoid traits are also associated with a wide spectrum of disorders, including schizophrenia (51). Therefore, despite correlations to delayed BD onset, these profiles should not be considered benign.

Regarding Character profiles and BD onset, the sCT (Cyclothymic) and sct (Melancholic) profiles were significantly associated (applying a liberal Bonferroni correction) with earlier onset in total BD, with the former retaining that significance in BDI and the latter in BDII. These profiles indicate mood dysregulation, as their name reflects. Cyclothymia, while distinct from BD due to less severe mood swings, shares genetic liability with BD, with some even viewing it within the broader bipolar spectrum (52). Melancholia is a specifier for severe depression in DSM – 5. Therefore, BDII, which has more genetic overlap than BDI with depression (15), may start earlier when melancholic traits are present. Concludingly, our findings highlight the impact of subaffective (cyclothymic and melancholic) personality variants on BD onset in accordance with previous literature (19, 53, 54).

Conversely, Sct (Bossy) related to later onset across groups, followed by SCt (Organized) in BD and BDI, and SCT (Creative) in BD, BDII and nominally in BDI. SCt (Organized) is considered a benign Character profile, suggestive of healthy functioning (35). Interestingly, although high SD and CO are protective for BD while high ST increases BD risk (30), the SCT (Creative) profile with high scores across all Character dimensions was protective regarding BD onset, reflecting how TCI profiles capture complex interactions not reflected in dimensions. Therefore, ST, considered a complex transpersonal construct, may offer resilience when high and paired with high SD and CO (SCT/Creative Character profile), but could also be associated with psychosis when the other Character dimensions are low (scT/Disorganized or Schizotypal Character profile) (35, 55–57). Notably, the protective effect of the SCT (Creative) profile on BD onset should be considered vis-à-vis the established genetic and phenotypical connection between BD and creativity (35, 56, 58–61).

While BDI findings generally aligned with overall BD findings regarding profiles, BDII analyses yielded some conflicting results. Notably, the nHR (Avoidant) and nhr (Schizoid) Temperament profiles and the SCt (Organized) Character profile were associated with disease onset in opposite directions in BDII compared to BD/BDI. A possible explanation could be the smaller BDII sample affecting statistical power. However, BDII is considered discrete from BDI, suggesting this conflict might reflect true differences between BD subtypes. For example, BDII has been shown to bear a closer genetic resemblance than BDI to Major Depressive Disorder (15).

Our results suggest that analyzing TCI dimensions in a vacuum may not sufficiently predict BD onset, while interactions of dimensions within cubes, reflected in TCI profiles, may offer a more informative and nuanced approach. Due to our control sample, the association between profiles and BD occurrence could not be explored. Lastly, it should be repeated that all results concerning TCI profiles in regard to BD onset were either significant, or nominally significant applying only the more liberal Bonferroni approach. None of these results were retained after applying stricter corrections. Therefore, all aforementioned results concerning these profiles should be considered exploratory and approached with caution.

Importantly, if Temperament and Character are causally associated with BD onset, it would be worth exploring potential clinical applications of our findings. Unaffected samples with other recognized risk factors for BD, such as substance use, positive family history of major psychiatric disorders or history of abuse, might benefit from additional screening for TCI dimensions or profiles to enhance risk assessment; those identified to be at higher risk could be prioritized for closer follow-ups or personalized preventive interventions. Furthermore, TCI could aid in differential diagnosis or predicting illness course. For example, BD displays higher NS and ST and lower HA than MDD (30). Therefore, if a patient with a history of depressive episodes has a TCI phenotype that is closer to BD than MDD, this could possibly suggest underlying BD diathesis and alert the physician to be cautious regarding the use of antidepressants. Therefore, it would be beneficial for clinicians to consider “temperamental predisposition” and personality in general when diagnosing and treating affective disorders (19, 23).

This study has several strengths. It is the first to use TCI profiles in association with BD onset age, allowing for more nuanced and accurate findings than analyses limited to TCI dimensions, taking into account complex interactions between same-cube dimensions. We cross-checked the associations with both linear and Cox regressions, with the former method proving more efficient. Thirdly, the sample size of this study was adequate at least for analyzing TCI dimensions. Additionally, to avoid mood symptoms interfering with personality assessment, all our patients had to be in stable euthymia ascertained by both physician judgment and objective mood assessment (HDRS, YMRS). Finally, out of various clinicodemographic data collected, we carefully selected our confounders to predate outcome (BD occurrence or onset) or even exposure (personality, considered formed by the beginning of adulthood), avoiding the inclusion of potential mediators in the pathway from exposure to outcome.

Certain limitations nevertheless also exist. Our findings may be prone to selection bias for two reasons. Control participants were a convenience sample recruited via snowball and not random sampling and were required to have no family history of major psychiatric disorder, therefore being non-representative of the general population. In a recent meta-analysis, unaffected patients’ siblings scored higher on HA and lower on SD than population controls (30). Therefore, our results may be more pronounced than expected in the general population. Additionally, personality may have affected control participants’ choice to voluntarily join the study. Moreover, patient participants came from a single, tertiary-care center and most had been previously hospitalized for a mood episode, therefore potentially being non-representative of the entirety of BD patients. All these reasons limit the generalizability of our findings if applied to the broader BD population compared to the general population.

Furthermore, personality in patients was assessed at recruitment, after BD onset, and assumed to be a proxy for personality traits at early adulthood. The underlying hypothesis is that personality remained relatively stable after BD onset and through the course of the disease. Although lifetime changes of TCI dimensions in BD have not been investigated, BD patients’ stability of FFM dimensions rivaled that of controls in a longitudinal study (62). Still, multiple relapses, persistent mood states and treatment might not leave personality intact; therefore, some degree of reverse causality (BD course altering certain personality aspects) and trait-state confounding cannot be excluded, and our results should be interpreted with caution. Future case-control studies including only incident cases (with minimal disease duration) rather than prevalent cases (as in our study) or, ideally, large population cohort studies would determine the causal effect of personality on disease onset much more safely.

The selection of confounders in our multivariate models deserves an additional mention. As no matching was applied between patients and controls, the two groups differed on age and educational attainment, which were statistically adjusted for in our models, as well as family and employment status. These last two variables were not included as confounders following our causal perspective, as they were assessed after the outcome (BD onset) and could hence not serve as its cause. However, it is unknown whether these well-documented BD-related impairments in personal, family and professional functioning (63) could also have impacted personality assessment at recruitment. Regarding age, this variable was included in case-control analyses as it was associated with both TCI dimensions and BD diagnosis in the total sample. However, age was excluded from case-only analyses as it was not associated with TCI dimensions in the patient sample while its association with AAO was primarily considered ‘non-causal’, an artefact of recruitment. Age was reported highly correlated with AAO in several BD studies yet not adjusted for in multivariate analyses of AAO (3, 64). The correlation is maximal with incident cases, as both ages coincide. However, other explanations of age – AAO correlation have also been proposed, including recall bias, as well as cohort (year of birth) effects, with patients in more recent birth cohorts reporting earlier onsets (65). Lastly, residual confounding cannot be ruled out in this observational study.

Finally, the analyses regarding TCI profiles were underpowered. To reduce error in multiple comparisons between profiles, two levels of Bonferroni corrections were applied. While strict corrections yielded no significant results, more liberal thresholds revealed promising findings, which should, however, be treated as merely exploratory and approached with caution but might benefit from future research with larger samples.

## 5 Conclusion

This study aimed to investigate associations of personality (Temperament and Character) with BD’s occurrence and AAO. While associations between TCI dimensions and BD had been reported in the past, this study uniquely implemented TCI profiles in the analyses conducted. The results largely align with existing literature regarding BD occurrence. Notably, this study identified, for the first time, significant links between specific TCI dimensions and the AAO of BD and subtypes. The inclusion of profiles suggested patterns that would likely remain concealed if only dimensions were considered. Despite its strengths, the study has limitations that call for further research and large multicenter cohort studies with emphasis on TCI profiles, which offer a more comprehensive approach to personality compared to dimensions. Overall, recognizing personality as a potential risk factor for BD would allow at-risk groups to receive timely, intensive, and personalized care, like more regular appointments, symptom assessment and medical treatment if required.

## Data Availability

The raw data supporting the conclusions of this article will be made available by the authors, without undue reservation.
