# Supplementary material for: Temperament and character traits and profiles: impact on bipolar disorder risk and onset age
Source: Front Psychiatry. 2026 Jan 6;16:1721514. doi: 10.3389/fpsyt.2025.1721514 (PMC12815727; doi:10.3389/fpsyt.2025.1721514)
Supplement: Supplementary file 1 [file Table1.docx]

**Supplementary Table 1:** Comparison of this study’s BD patients and Healthy Controls samples with Vitoratou et al. (2015) General Population sample.

|  | BD Patients Sample  (A, N=179) | Healthy Controls Sample  (B, N=96) | General Population sample  (C, N=306) | Statistics  A vs. C | Statistics  B vs. C |
| --- | --- | --- | --- | --- | --- |
| Male Sex | 67 (37.43%) | 35 (36.46%) | 142 (46.41%) | Chi2=3.71, p=0.054 | Chi2=2.93, p=0.087 |
| Age (years) | 47.99 (12.01) | 40.36 (12.61) | 49.04 (10.91) | t=0.985, p=0.325 | t=6.545, p<0.001 |

Vitoratou S, Ntzoufras I, Theleritis C, Smyrnis N, Stefanis NC. Temperament and character dimensions assessed in general population, in individuals with psychoactive substance dependence and in young male conscripts. Eur Psychiatry. (2015) 30:474-9. doi: 10.1016/j.eurpsy.2015.01.007

**Supplementary Material:** Detailed description of the Temperament and Character profiles as proposed by Cloninger

**Temperament profiles**

The **NHR** profile, dubbed as “Sensitive” or “Narcissistic”, relates to a strong need for approval and vulnerability to perceived failure. **NhR** is the “Passionate” or “Histrionic” profile, marked by attention seeking and overestimation of emotional bonds. **NHr**, known as “Explosive” or “Borderline”, relates to emotional and identity instability and self-harming behavior. **Nhr**, dubbed as “Adventurous” or “Antisocial”, refers to a personality disorder (PD) characterized by low empathy, aggression and rule violation for personal benefit. **nHR**, though called “Cautious”, actually reflects traits of Avoidant PD, such as social avoidance, overwhelming anxiety and feelings of shame. **nHr**, dubbed as “Methodical” or “Obsessional”, is associated with obsessive compulsive traits. **nhR**, referred to as “Slaid” or “Reliable”, is considered a “safe” profile not typically associated with psychopathology. Finally, the **nhr** profile, known as “Indifferent” or “Schizoid”, reflects traits of a schizoid personality like voluntary social withdrawal. Profiles linked to high NS appear to be associated with “emotional” PDs previously known as “Cluster B”, characterized by emotional volatility and unpredictability, like the Narcissistic, Histrionic, Antisocial and Borderline PDs.

**Character profiles**

The **SCT** profile is called “Creative” or “Enlightened” and corresponds to high self-esteem, a sense of unity, spirituality and hyperthymic affect. **SCt**, labeled “Organized”, reflects good adjustment and is considered protective. In contrast, **ScT** is known as “Absolutist” or “Paranoid” or “Fanatical”, reflecting a high sense of self, distrust of others, rigid beliefs and misinterpretation of others’ behaviors. The **sCT** profile is dubbed “Moody” or “Cyclothymic” and characterized by mood lability. The **Sct** profile is called “Bossy” or “Authoritarian”/ “Autocratic”, predicting leadership traits (high SD), but also oppressive qualities (low CO). **sCt** or “Dependent” describes low self-confidence and over-reliance on relationships for security. **scT**, known as “Disorganized” or “Schizotypal”, involves a strong belief system but weak sense of self and community, often resulting in paradoxical or bizarre behavior. Finally, **sct**, the “Apathetic”, “Downcast” or “Melancholic” profile, is characterized by low mood and passivity.

References

Cloninger CR, Svrakic DM, Przybeck TR. A psychobiological model of temperament and character. Arch Gen Psychiatry. (1993) 50:975-90. doi: 10.1001/archpsyc.1993.01820240059008

Cloninger CR. Feeling good: The science of well-being. New York, NY, US: Oxford University Press (2004).

Cloninger CR. The temperament and character inventory (TCI) : a guide to its development and use. 1st ed. St. Louis, MO, US: Center for Psychobiology of Personality, Washington University (1994).
